# The Influence of Coral Reef Benthic Condition on Associated Fish Assemblages

**DOI:** 10.1371/journal.pone.0042167

**Published:** 2012-08-01

**Authors:** Karen M. Chong-Seng, Thomas D. Mannering, Morgan S. Pratchett, David R. Bellwood, Nicholas A. J. Graham

**Affiliations:** 1 Australian Research Council Centre of Excellence for Coral Reef Studies, James Cook University, Townsville, Queensland, Australia; 2 School of Marine and Tropical Biology, James Cook University, Townsville, Queensland, Australia; Swansea University, United Kingdom

## Abstract

Accumulative disturbances can erode a coral reef’s resilience, often leading to replacement of scleractinian corals by macroalgae or other non-coral organisms. These degraded reef systems have been mostly described based on changes in the composition of the reef benthos, and there is little understanding of how such changes are influenced by, and in turn influence, other components of the reef ecosystem. This study investigated the spatial variation in benthic communities on fringing reefs around the inner Seychelles islands. Specifically, relationships between benthic composition and the underlying substrata, as well as the associated fish assemblages were assessed. High variability in benthic composition was found among reefs, with a gradient from high coral cover (up to 58%) and high structural complexity to high macroalgae cover (up to 95%) and low structural complexity at the extremes. This gradient was associated with declining species richness of fishes, reduced diversity of fish functional groups, and lower abundance of corallivorous fishes. There were no reciprocal increases in herbivorous fish abundances, and relationships with other fish functional groups and total fish abundance were weak. Reefs grouping at the extremes of complex coral habitats or low-complexity macroalgal habitats displayed markedly different fish communities, with only two species of benthic invertebrate feeding fishes in greater abundance in the macroalgal habitat. These results have negative implications for the continuation of many coral reef ecosystem processes and services if more reefs shift to extreme degraded conditions dominated by macroalgae.

## Introduction

An ecosystem’s ability to recover from degradation is eroded by increases in frequency, intensity and array of disturbances [1]–[4]. On coral reefs, increasing anthropogenic pressures (e.g. fisheries exploitation) and climate change, are compounding upon pre-existing disturbances (e.g. cyclones) and causing declines in coral cover and structural complexity [5], [6], associated changes in coral and fish community composition [7]–[9], and shifts in the dominant benthic biota [10]–[12]. Documented shifts on coral reefs include changes to corallimorphs, sponges, or most often, macroalgae domination of the benthos [10]–[12]. Although these other benthic lifeforms are typical components of most reefs, scleractinian coral domination is considered preferable; corals function as the main provider of the complex structural habitat that is largely responsible for the high diversity of reef associated organisms, and the provision of a range of ecosystem services, such as vital food resources [13]–[15].

All major coral reef regions of the world have undergone declines in coral cover [5], [16], [17]. In conjunction with these reductions in coral cover, is an increasing documentation of shifts in the dominant benthic biota (reviewed by: [12]) that focus primarily on causes of the shifts, and subsequent changes in the benthic community composition. For example, although the causes attributed to the shift from coral to macroalgae on Jamaican coral reefs included overfishing of herbivorous fish, hurricane Allen and disease mediated collapse of urchin populations, the description was based solely on benthic composition [11]. How these changing benthic communities interact with underlying substrata, or influence the rest of the coral reef ecosystem, for example reef fish assemblages, is poorly understood.

Complex interconnections among organisms and with their physical environment, imply that changes to one aspect of the ecosystem may lead to a subsequent series of, often unanticipated, changes to the ecosystem’s community assemblage [18]–[20]. Strong relationships exist between coral reef fishes and their habitat [21], [22], although there is variability in the specific responses of different fishes, and of different ontogenetic stages, to changes in coral cover [9], [23], [24]. Live coral loss can trigger shifts in the entire fish assemblage [25], [26], and prompt declines in abundance and diversity of fishes [27], [28]. The potential for other benthic organisms to provide the necessary habitat for reef fishes has not been widely investigated, although Syms and Jones [29] showed that soft coral was not a favourable habitat replacement for hard corals. From non-marine ecosystems it appears possible that some organisms may provide habitat for an equally, or more diverse community, or alternatively, that changes in the habitat-providing organisms can be detrimental to diversity. As an example of the former, Brazilian forests contained 26 lizard species whereas the grassland alternative contained 30 species [30]. In contrast, lakes lose their high submerged macrophyte and animal diversity following shifts to turbid eutrophic waters [31], while shifts from rangelands to desert lead to much reduced diversity [32].

The interactions between the foundational structure upon which the live reef is built, the underlying substratum, and changes in the benthic community, may hinder essential ecosystem processes required for recovery, and perpetuate an alternative community. For example, coral recruit survivorship is considered an essential process for recovery [33], [34] and can be inhibited by burial and damage of new recruits by highly mobile rubble substrata during storms [35]–[37]. The relationships between a reef’s underlying substratum and dominant benthos are generally unknown, but knowledge of such relationships would further our understanding of the development and endurance of degraded conditions on coral reefs.

Coral reefs of the Seychelles archipelago offer a unique opportunity to assess differing benthic communities. The inner Seychelles islands are geographically isolated, were severely impacted by the 1998 mass bleaching event, and there is a good record of post-disturbance degradation [6], [17], [38], [39]. Ten years after this major bleaching event, coral cover in the inner Seychelles ranged from <5% coral cover to >20% coral cover, which is amongst the lowest in the region [6], [40]. Individual reefs have shown highly varied responses to disturbance, and there have been reports of benthic community shifts on some reefs [28], [40]. However, detailed characterisation of the benthic condition of these reefs is lacking, along with the implications of benthic condition for other aspects of the reef community. We therefore quantitatively characterised the benthos, underlying substratum, and fishes of inner Seychelles reefs to investigate: 1) if there was a link between underlying substrata and benthic condition; and 2) the relationship between benthic condition and the taxonomic and functional composition of associated fish assemblages.

## Materials and Methods

### Ethics Statement

A research permit for this work was granted by the Seychelles government through the Seychelles Bureau of Standards; permit number A0347.

### Study Site and Sampling Design

Twenty-one carbonate fringing reefs within a 3600 km^2^ area around the inner Seychelles islands (4 30′S, 55 30′E) were surveyed in October 2010. Fishing practices in the inner Seychelles use non-destructive techniques (handlines, traps and octopus harpooning are the most widely used; [41]), and there is relatively low variability in fishing pressure along the shallow fringing reefs among the islands, with most fishing occurring in deeper water [42], [43]. At each reef, four 50 m transects were laid at approximately 4 m depth, perpendicular to the reef slope. The following data were collected along each transect; 1) live benthic cover recorded at 0.5 m intervals, 2) underlying substratum quantified at 0.5 m intervals, 3) number and identity of all fish greater than 8 cm were recorded along a 5 m wide belt (to minimise disturbance, large, mobile species were counted as the transect was laid; [44]), and 4) structural complexity was recorded using both a 6-point scale and by estimating the number of small refuge holes, <10 cm diameter, along two 10×1 m sub-transects (following [45]). Scleractinian corals and macroalgae were identified to genus and/or morphological group, while other algae were identified to functional group. Other benthic organisms recorded included corallimorphs, sponges and zoanthids. For analyses, branching acroporids, massive *Porites*, and favids were differentiated from the rest of the coral genera (grouped as ‘other hard corals’) due to their high coverage. The underlying substratum, defined as the substratum below recorded benthic cover or the top 10 mm of sand/sediment, was categorised into loose dead coral rubble, consolidated rubble (rubble pieces that were showing visual and tactile signs of amalgamation), solid carbonate pavement, or bommie (isolated coral outcrops). Fish species were assigned to 8 functional groups based on the literature and FishBase: obligate corallivores, browsing herbivores, other herbivores (including scrapers, grazers, excavators, detritivores), planktivores, piscivores, non-coral invertivores (hereafter invertivores), omnivores (consume animal and plant material) and generalist carnivores (fish and invertebrate feeders). Additionally, the level of exploitation sustained by different fish species was assigned at four levels: primary targets, important by-catch, occasional by-catch and non-fished species [46].

**Figure 1:**
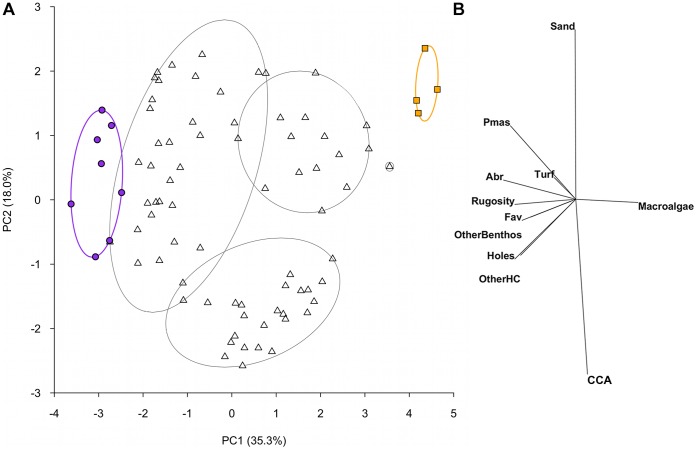
Principal components analysis of benthic habitat variables. (A) Spatial variation in benthic habitat on reefs at the transect level, shown for the first two components from a principal components analysis on natural log(x+1) transformed and normalised data. Ellipses show groupings calculated from a slice taken through a hierarchical cluster analysis at a Euclidean distance value of 4. Data symbols represent transects within reefs; filled circles and squares highlight transects within the extreme clusters for visualisation purposes. Purple circles and ellipse shows high complexity coral cluster consisting of 8 transects from 2 reefs; orange squares and ellipse shows low-complexity, high macroalgae cluster consisting of 4 transects from 1 reef; triangles are transects that fall within intermediate clusters. (B) The relative contribution of the 11 benthic habitat categories to the observed variation in reef benthic condition. Pmas – massive *Porites*; Abr – branching *Acropora*; Fav – favids; OtherBenthos – non-coral or algae benthic organisms; OtherHC – all other scleractinian corals; CCA – crustose coralline algae.

### Analyses

The data were organized into four matrices; i) benthic habitat (11 variables; including the two complexity measures) that was natural log transformed to improve the spread of the data, and normalised to standardize the contribution of variables measured as percent cover and those measured on different scales, ii) underlying substrata cover (4 variables), iii) fish functional group abundances (8 variables) that were square-root transformed to downweigh abundant groups [47], and iv) fish species abundances (152 species) that were also square-root transformed to downplay the influence of highly abundant species. The complexity measures were included with the benthic cover variables because these measures are thought, at least in part, to reflect the complexity provided by live benthic organisms (e.g. [6], [48], [49]). Within- and among-reef variation was assessed using ordination methods on dissimilarity matrices in the statistical software PRIMER; correlation-based principal components analysis (PCA) on Euclidean distances for the underlying substratum and benthic cover matrices (as the data is continuous and needed to be normalised; [47]), and non-metric multidimensional scaling (nMDS) on Bray-Curtis dissimilarities to account for high zero counts [47], for the fish matrices. Pairwise relationships between all variables within a matrix showed no collinearity (r<0.7; [50]). Groupings in the benthic cover PCA were assessed by overlaying slices from a hierarchical cluster analysis using group averaging of the same Euclidian distance matrix.

**Figure 2:**
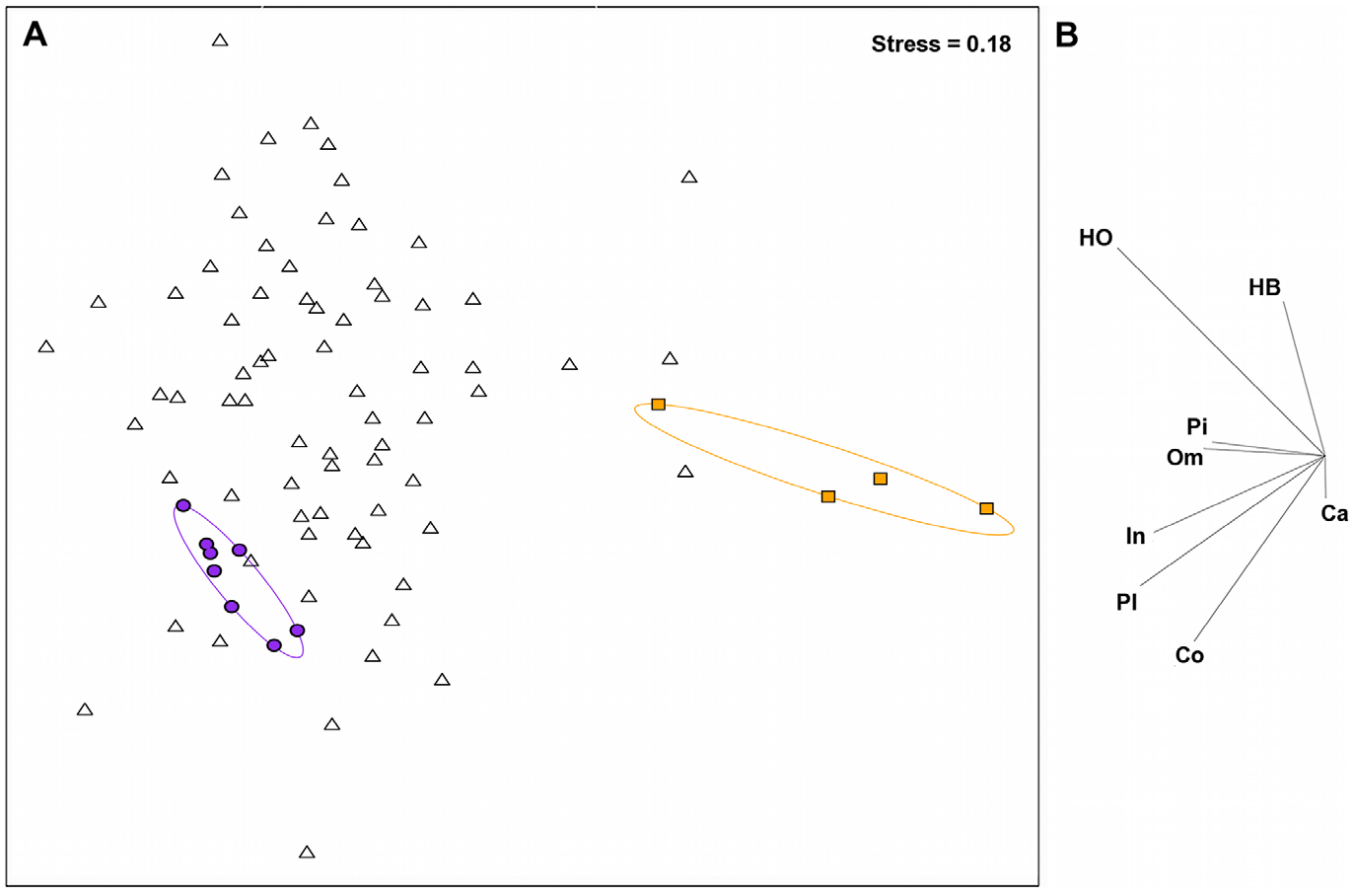
Non-metric multidimensional scaling analysis of fish functional groups. (A) Spatial variation in the reef fish functional group abundances on reefs at the transect level, assessed using a non-metric multidimensional scaling plot on square-root transformed data. Data symbols represent transects within reefs. For visualisation purposes, filled circles and squares, and ellipses highlight the transects within the extreme clusters calculated from a slice taken through the Benthic data’s hierarchical cluster analysis at a Euclidean distance value of 4. Purple circles and ellipse shows high complexity coral cluster, orange squares and ellipse shows low-complexity, high macroalgae cluster. (B) The relative contribution of the 8 fish functional groups to the observed variation on reefs. HB – browsing herbivores; HO – non-browsing herbivores; Pi – piscivores; Om – omnivores; In – non-coral invertivores; Pl – planktivores; Co – obligate corallivores; Ca – generalist carnivores.

**Table 1:** Fish taxa contributing to the similarity within, and dissimilarity between, the extreme groups of low-complexity macroalgae and complex coral.

			Similarity		Dissimilarity
Species	FG	FP	Macroalgae (49.5%)	Coral (46.8%)	(88.7%)
*Lethrinus harak*	Ca	I	35.69		2.67
*Cheilio inermis*	Ca	N	30.91		3.21
*Thalassoma herbraicum*	In	N	14.68	6.02	2.11
*Chromis atripectoralis*	Pl	N		9.75	5.45
*Chlorurus sordidus*	HO	P		8.95	4.12
*Chaetodon trifasciatus*	Co	N		8.19	4.42
*Plectroglyphidodon lacrymatus*	HO	N		5.43	3.87
*Pomacentrus sulfureus*	Pl	N		5.29	3.92
*Gomphosus caeruleus*	In	O		5.09	3.11
*Ambyglyphidodon leucogaster*	Pl	N		4.08	3.33
*Cheilinus trilobatus*	In	I		4.03	1.63
*Labroides dimidiatus*	In	N		3.69	2.30
*Scarus niger*	HO	I		3.26	2.68
*Halichoeres marginatus*	In	O		2.73	2.23
*Ctenochaetus striatus*	HO	I		2.70	2.21
*Halichoeres hortulanus*	In	O		2.70	2.05
*Pomacentrus trilineatus*	Pl	N			2.24
Carangidae	Pi	P			2.00
*Hemigymnus fasciatus*	In	O			1.76
*Halichoeres nebulosus*	In	O			1.69
*Zanclus cornutus*	In	O			1.55
*Stethojulis albovittata*	In	O			1.53
*Labrichthys unilineatus*	Co	O			1.44
*Macropharyngodon bipartitus*	In	O			1.40
*Oxymonacanthus longirostris*	Co	N			1.32
*Centropyge multispinis*	In	O			1.30
*Scolopsis frenatus*	In	O			1.27
*Lethrinus obsoletus*	Ca	I			1.24
*Chromis ternatensis*	Pl	N			1.18
*Zebrasoma scopas*	HO	N			1.11
TOTAL % contribution			81.28	71.91	70.33

SIMPER analysis performed on square-root transformed abundance data. Cutoff for low contributions: 70%. Average similarity or dissimilarity reported in parentheses. Functional group (FG) acronyms defined in Figure 2 legend. Fishing pressure (FP) exerted on the species. P – primary target; I – important by-catch; O – occasional by-catch; N – not targeted.

### Relationship between Data Matrices

Variability in benthic composition among reefs was related to underlying substratum, and also the composition of fish assemblages, in two ways. First, data points ( = transects) on the underlying substratum and fish assemblage ordinations were colour-coded according to groups identified from the benthic cover hierarchical cluster analysis to visualize relationships. Second, the BEST BIO-ENV routine was carried out using a Spearman rank correlation between the different similarity resemblance matrices to identify the variable or group of variables that best explained similarities among the data matrices [47]. The overall significance of the BEST routine was assessed using a permutation test under the null hypothesis of no linkage of variables between matrices (maximum permutations  = 999; [51]).

### Comparing Variables along a Gradient of Contrasting Benthic States

A combination of cluster analysis and ordination showed the presence of contrasting benthic assemblages along a gradient from coral to macroalgae. To investigate whether there were any fish species that typified either assemblage, we ran a similarity of percentages (SIMPER) analysis using a subset of the fish species matrix that reflected the two extreme clusters of transects identified by the slice through the benthic cluster diagram. This represented transects dominated by macroalgae versus transects with high coral cover and structural complexity. An index of the fish functional group diversity was calculated using the Shannon-Weiner diversity index, *H’*, which takes into account both abundance and the number of functional groups (maximum n = 8). The relationships between the benthic gradient (the benthic PCA’s first principal component) and fish functional group diversity (*H’*), fish species richness, total fish abundance, and individual functional group abundances were examined using General Additive Models (GAM). GAMs incorporate the possibility of non-linear relationships between the response and predictive variables [50]. Reef was included as a random effect variable using restricted maximum likelihood estimation (REML) using the gam and gamm functions of the mgcv package in R.

**Figure 3:**
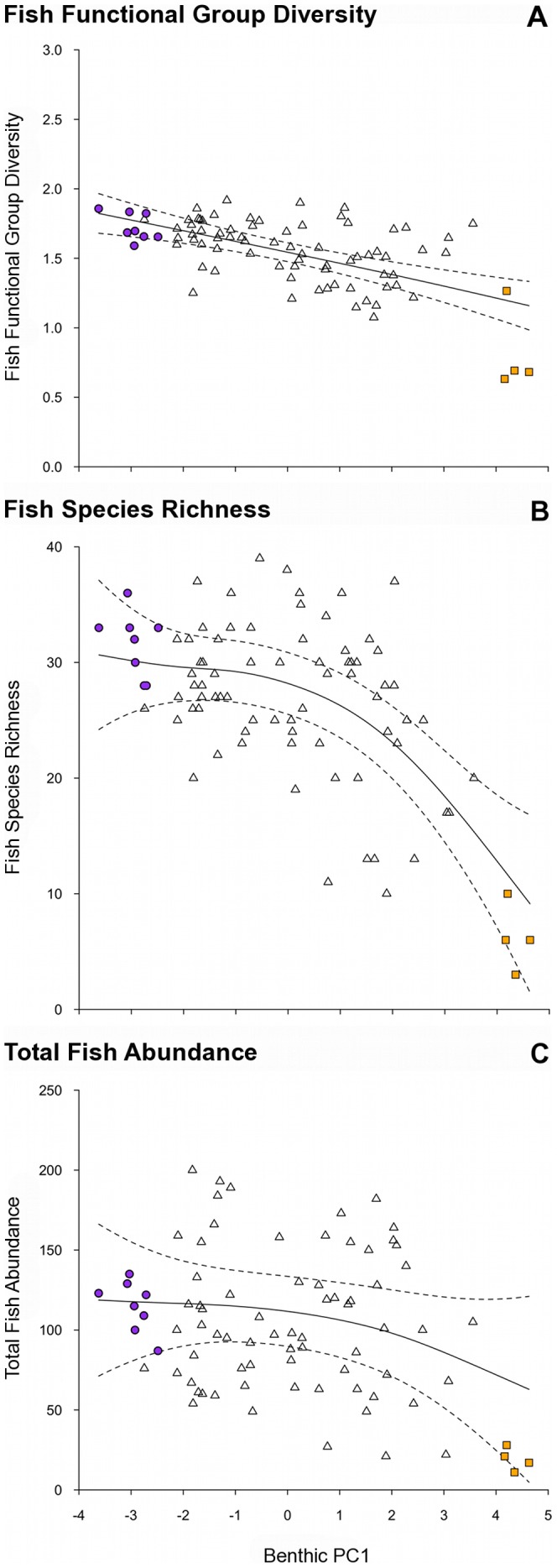
Relationships between the gradient in benthic habitat condition and fish assemblage metrics. Benthic habitat condition (PC1 axis): negative values – complex coral habitats; high values – low-complexity macroalgae habitats. Fish assemblage metrics: (a) fish functional group diversity (an index calculated using the Shannon-Weiner diversity index (H’) at the functional group level), (b) fish species richness, (c) total fish abundance. Plotted are fitted parameter estimates ±95% confidence intervals based on GAM with Reef as a random variable. Note that (c) represents a statistically non-significant relationship. Two extreme outliers were excluded from (C) to aid visual representation, but were included in the analysis. Symbols as in previous figures. Note different scales along y-axis.

**Table 2:** Results of generalized additive mixed models (GAMM) used to model response variables with respect to the gradient in benthic habitat (Benthic PC1), with Reef as a random variable.

Response variable	*df*	*F*	*p*	*r^2^*
Fish functional group diversity	1.15	26.024	**	0.375
Fish species richness	2.687	27.135	**	0.434
Total fish abundance	1.642	3.133	NS	0.081
Obligate corallivores	7.546	26.938	**	0.749
Browsing herbivores	1	2.789	*	0.066
Non-browsing herbivores	2.226	1.756	NS	0.094
Non-coral invertivores	1.775	4.963	*	0.139
Generalist carnivores	1	0.002	NS	−0.012
Omnivores	2.336	3.593	NS	0.104
Piscivores	1	3.523	NS	0.05
Planktivores	1	2.127	NS	0.08

*df*: estimated degrees of freedom for smooth term (Benthic PC1; 1 =  linear).

*p*: **p<0.001, *p<0.05, NS *p*>0.05.

*r^2^*: proportion of variation explained by the benthic habitat gradient (negative value  =  model is a worse representation than the Null model).

**Figure 4:**
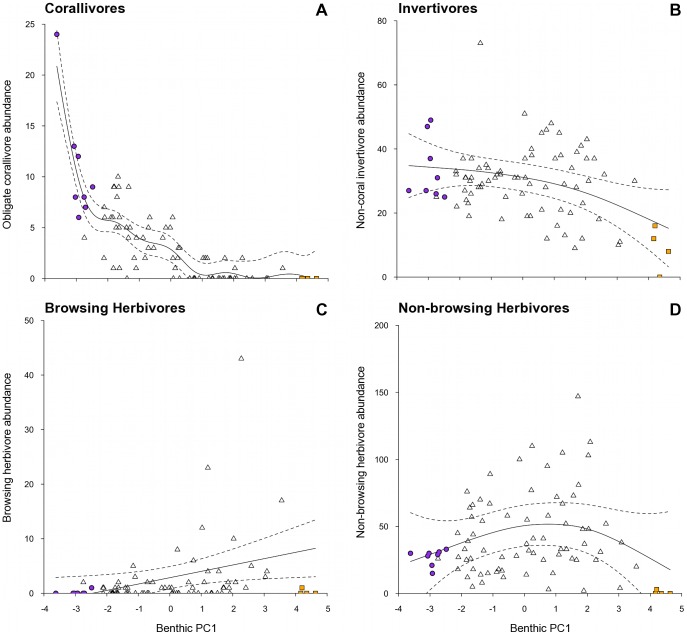
Relationships between the gradient in benthic habitat condition and abundances of fish functional groups. Benthic habitat condition (PC1 axis): low values – complex coral habitats; high values – low-complexity macroalgae habitats. Abundances of fish functional groups: (a) obligate corallivores, (b) non-coral invertivores, (c) browsing herbivores, and (d) non-browsing herbivores. Plotted are fitted parameter estimates ±95% confidence intervals based on GAM with Reef as a random variable. Note that (d) represents a statistically non-significant relationship. An extreme outlier was excluded to aid visual representation from (B), (C), and (D), but was included in the analyses. Symbols as in previous figures. Note different scales along y-axis.

## Results

### Benthic Reef Assemblages

Benthic cover of reef organisms was highly variable among the 21 reefs in the inner Seychelles. Live coral cover ranged from 0 to 47% (±5.1 SE) and macroalgae cover from 0 to 76% (±6.7 SE) per reef (Figure S1). The first principal components axis (PC1) of the benthic PCA differentiated transects along a gradient from high coral cover (up to 58% per transect) and structural complexity (rugosity score up to 4, and up to 1150 10 cm holes) at negative PC1 scores, to high macroalgae cover (up to 95%) and low structural complexity (rugosity score down to 0.5, and as few as 30 10 cm holes) at positive PC1 scores (Figure 1). A separation from sand and sediment-laden turf to crustose coralline algae was represented by PC2. A slice through a cluster analysis at a Euclidean distance of 4 represented six groupings in the data, including two groups at extreme ends of PC1, and four intermediate groups (Figure 1).

### Underlying Substrata

The underlying substrata of the reefs varied from loose rubble to consolidated carbonate pavement. When highlighted on the underlying substrate PCA plot, transect groupings from the benthic cluster analysis were not apparent, however reefs found at both extreme ends of the benthic PC1 were associated with more stable substrata. It is to be noted that the stress level of the MDS was fairly high, so although general patterns are robust, details need to be interpreted with some caution [47]. A BEST analysis (r_s_ = 0.16, p<0.05) corroborated this pattern, finding a weak but significant correlation between the benthic and underlying substrata distance matrices, specifying the presence of pavement rather than rubble as the principal cause of similarity.

### Fish Assemblages

A total of 152 fish species were recorded from the study site, with 3 to 38 species observed per transect. All of the eight fish functional groups were more strongly associated with transects plotted on the left hand side of the MDS plot (Figure 2). Highlighting the transect clusters found by the benthic analysis, on the fish functional group MDS plot indicated that the more fish-depauperate reefs corresponded to reefs with the highest levels of macroalgae (BEST r_s_ = 0.48, p<0.001). Fourteen fish species, including planktivores, invertivores, an obligate corallivore and non-browsing herbivores (a bioeroder, a scraper and two detritivores) contributed to 70% of the similarity within the cluster of transects at the high coral cover, high complexity end (herein referred to as complex coral habitats) of the benthic PC1 (Table 1). These 14 species included a primary fishery target species, *Chlorurus sordidus*, and 3 important- and 3 occasional fishery by-catch species (Table 1). In comparison, only 3 species – *Thalassoma herbraicum* (an invertivore), *Cheilio inermis* and *Lethrinus harak* (both generalist carnivores, and the latter is an important fishery by-catch species), contributed to 70% of the similarity within the cluster of transects at the high macroalgae cover, low-complexity end (herein referred to as low-complexity macroalgae habitats) of the benthic PC1 (Table 1). One species, *T. herbraicum* was common to both groups. Sixteen species explained 49.3% of the dissimilarity between the complex coral and low-complexity macroalgae habitats and represent 5 of the 8 defined functional groups (Table 1).

The transition along the benthic gradient from complex coral to low-complexity macroalgae associated with PC1 (Figure 1), corresponded with a decline in fish functional group diversity (r^2^ = 0.375, p<0.001; Figure 3a), overall fish species richness (r^2^ = 0.434, p<0.001; Figure 3b) but not total fish abundance (r^2^ = 0.081, p>0.05, Figure 3c) (Table 2). For the abundance of fish within functional groups, PC1 of the benthic PCA corresponded with a strong decline in obligate corallivore abundance (r^2^ = 0.754, p<0.001, Figure 4a), a weak decline in invertivore abundance (r^2^ = 0.139, p<0.05, Figure 4b), and a very weak increase in browsing herbivore abundance (r^2^ = 0.066, p<0.05, Figure 4c)(Table 2). No relationships were found between the benthic habitat gradient and the abundances of the other five fish functional groups (Table 2; non-browsing herbivorous species also Figure 4d).

## Discussion

This study found markedly different fish composition along a multivariate gradient of reef benthic conditions ranging from complex coral habitats to low-complexity macroalgae habitats. Very different fish assemblages were linked with the two habitat extremes, not only in terms of species present, but also richness and diversity at both species and functional group level. The strongest relationships with the habitat gradient were found at the overall fish assemblage scale, rather than at the individual functional group scales. Obligate corallivorous fishes were the exception, and are known for their dependence upon live corals [52]. The dependence of reef fish assemblages on the coral reef benthos has been demonstrated through numerous before-after studies of fish and benthic changes through disturbance events (reviewed by: [9], [23]). In contrast, this study assesses the role of a broad array of benthic conditions following disturbance, on reef fish assemblages, providing useful insights into potential future compositions of reef fishes.

At the extreme ends of the benthic gradient, complex coral habitats support a higher number of fish species and functional groups than low-complexity habitats dominated by macroalgae. A major consequence for many ecosystems facing degradation is ecological homogenisation, whereby multiple specialist species or groups are replaced by fewer, more generalist species or groups leading to much simpler ecosystems [53], [54]. Our results appear to support this theory with the low-complexity macroalgae habitats lacking many of the more specialised coral reef fish functional groups (e.g. obligate corallivores and coral-associated planktivores; [45], [55]) and also the essential groups for the provision of key ecological processes (e.g. herbivores; [56]–[58]). While macroalgae provide 3-dimensional structure, in comparison to the often intricate and unyielding skeletal structures of scleractinian corals, it is a more homogeneous and flexible habitat that appears to be less favourable to reef fishes [59].

Macroalgal-dominated reefs have long been regarded as degraded reef states [11]. This study provides some empirical evidence that macroalgal-dominated reefs are unfavourable for the wider ecosystem’s ecological communities and economic potential. Nevertheless, habitats with abundant macroalgae can be naturally occurring and provide important refuges for juvenile reef-associated fishes [60]. Juvenile *Cheilio inermis* for example, are present only in *Sargassum* stands in Western Australia [60]. Also, *Sargassum* and *Turbinaria* algal stands have been present on Seychelles coral reefs for some time [61], [62], although the influence of human settlement on macroalgal presence is not known. Importantly, macroalgal cover has shown substantial expansion following the 1998 bleaching event [28] and is continuing to increase in cover [63]. Given the high cover of macroalgae documented in our study, and the habitats surveyed, it is likely that some of the sites represented recently degraded reef states. Our study suggests that expansion of macroalgae on reefs will have substantial negative repercussions for associated fish diversity.

Herbivores are considered the most important functional group of fish on coral reefs through their role in mediating the competition for space between corals and algae [57], [64], [65]. Indeed, negative relationships exist between herbivore biomass and macroalgae cover [66]–[69], although a distinction has been found between herbivorous species that maintain low algal biomass, and browsing species that will consume mature macroalgae thalli [58], [70]. Surprisingly therefore, there was no substantial increase in either of the two herbivorous functional groups along the benthic gradient found in this study. Similarly, a study of benthic changes across 7 countries in the Indian Ocean spanning the 1998 coral bleaching event found no increase in herbivore abundance in response to the increase in benthic space available for algal growth [6], while browsing species in Australia show no correlation with increasing macroalgal cover on the GBR [69] or Ningaloo reef [71]. Although browsing herbivores have been able to reverse phase shifts in small-scale experimental settings surrounded by intact reef [58], reefs with high fleshy macroalgal cover tend to have low functional redundancy amongst browsing herbivores [70], and dense macroalgal stands can inhibit herbivory [59]. Indeed, the ability of browsing herbivores to perform their vital function on macroalgae-dominated reef systems is very poorly understood.

The identified differences in the fish community with changing benthic condition are likely to have implications for ecosystem service provision [10], [72]–[74]. Major ecosystem services associated with reef fishes include the provision of fisheries and tourism [13], [75], [76]. Therefore, as fish species richness and functional group diversity decreases across the benthic condition gradient, the multi-species fishery and substantial dive tourism industry of the Seychelles are likely to be negatively affected by shifts away from complex coral-dominated reefs [41], [73], [77]. Specifically, there was a 5-fold difference in fish abundances at the benthic extremes of our study: macroalgal-dominated reefs had an average of 19.3 (±3.6 SE) fish per 250 m^2^ compared to 105.3 (±5.4 SE) fish per 250 m^2^ at the reef with the highest overall coral cover and complexity. Moreover, two of the five primary fishing target species, and 19 important by-catch species [46] were present on reefs with highest overall coral cover and complexity compared to no primary target species, and only one important by-catch species on low complexity and macroalgae-dominated reefs. This 5-fold difference in total fish abundance and the reduction in target species, is likely to reflect a decline in fishery potential. This contrasts with results from the Caribbean where macroalgae-dominated reefs appeared to sustain high fish species richness [78]. Similarly, studies of tourist preferences show that fish abundance and diversity play a major role in attracting and satisfying dive tourists (e.g. [79]–[81]).

Although we predicted that the stability of the underlying substratum would interact with the condition of the benthos, with stable substrata having higher coral cover than mobile rubble reefs, we found only weak relationships. Studies in rubble-dominant locations, such as former dynamite fishing areas, have found substantially lower coral cover on rubble versus stable rocky sites [35]. Furthermore, other macro-benthic organisms such as reef sponges have been found to have decreased growth rates on mobile rubble substrata compared to stable rock substrata [37]. Our results showed that many of the rubble dominated transects did have low coral cover (where rubble was >80%, mean coral cover was 4.9% (±1.9 SE)). However, many other transects that had little rubble also had low coral cover (22/46 transects with <5% rubble had <10% coral cover), indicating that substratum stability was not the only variable influencing coral cover. Interestingly however, both the coral-dominated and macroalgal-dominated extremes were associated with more stable substrata, suggesting that substratum stability is important in enabling these macrobenthic organisms to survive to maturity.

The multivariate gradient of benthic conditions found in this study indicates a continuum of reef states. However, in the absence of long-term time series data and/or experimental manipulations it is not possible to establish the stability of our extreme benthic state categories [82]. Similarly, the reefs in the middle of the continuum may be fairly stable in their own right, or in transition (i.e. degrading or recovering) between different characteristic equilibrium states because of various natural disturbances or perturbations [8], [32], [33], [83]. Regardless, it is clear that more degraded reefs, in terms of coral cover, diversity and structural complexity, host more depauperate reef fish assemblages.

As coral reefs continue to degrade due to a range of anthropogenic drivers, and alterations in community compositions occur, it is imperative that we understand how changes in one aspect of an ecosystem affect the rest of the ecosystem. From a management perspective, the fact that many reefs do not exist in discrete states means that few generalisations are possible, and reef specific data may be required to implement necessary management plans [32], [84]. While many previous studies have linked loss of fish diversity with loss of coral cover, the lack of reciprocal increases in herbivorous fishes to counter increases in algal cover is alarming, with negative implications for the continuation of many coral reef ecosystem processes and services if more reefs shift to macroalgal-dominated states. Clearly, prevention of further reef degradation through a reduction in anthropogenic pressures, is of critical importance because the repercussions of declining habitat condition may be far reaching.

## Supporting Information

Figure S1 **Proportional cover of benthic biota per site.**
(TIF)
