# Supplementary figures and images for: The Influence of Coral Reef Benthic Condition on Associated Fish Assemblages
Source: PLoS One. 2012 Aug 1;7(8):e42167. doi: 10.1371/journal.pone.0042167 (PMC3411644; doi:10.1371/journal.pone.0042167)

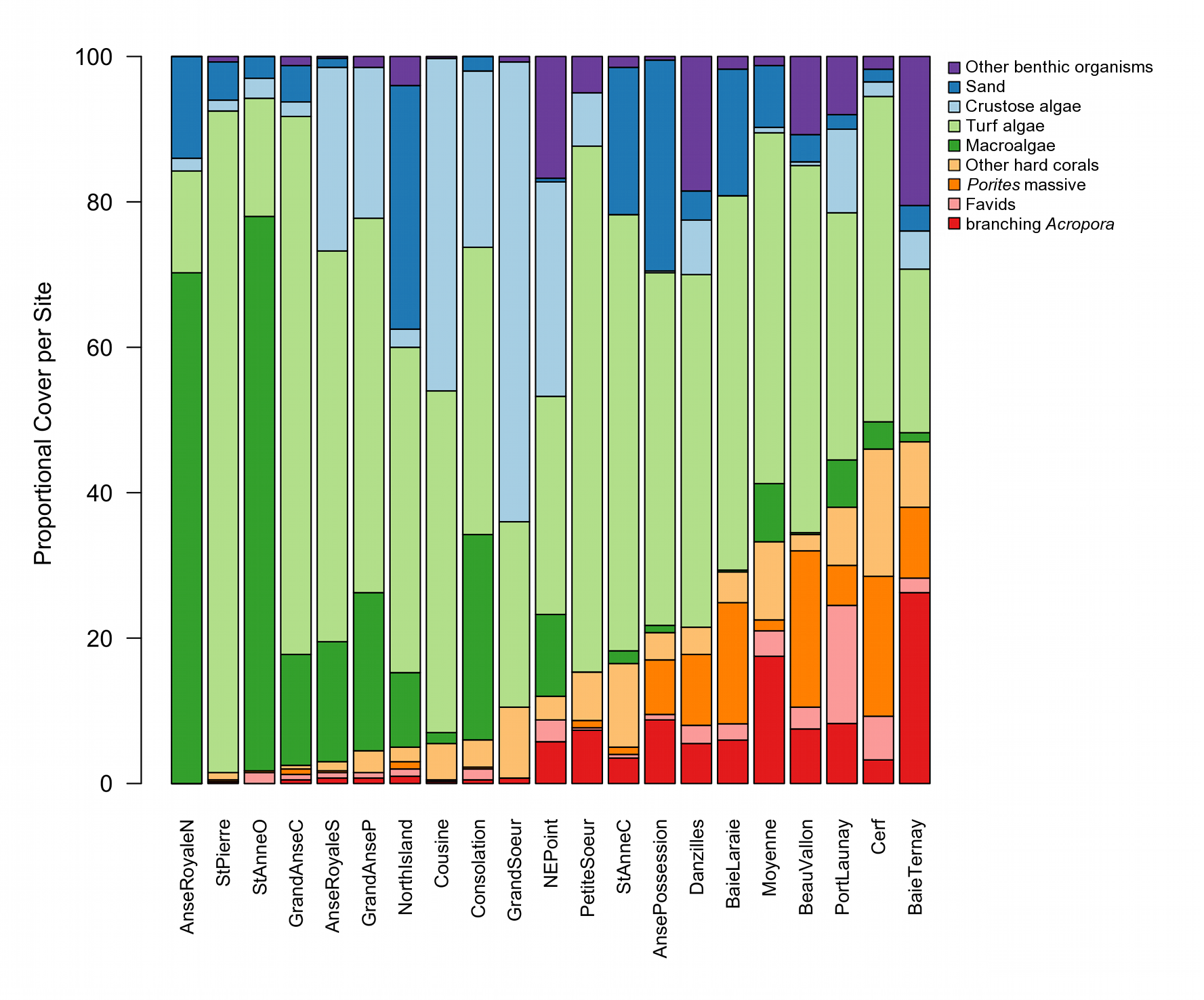

Supplement: Figure S1 — Proportional cover of benthic biota per site. (TIF) [file pone.0042167.s001.tif]
